# From the First Tooth to the Intensive Care Unit: Diet, Gut Microbiota and Neuroinflammation Across the Life Course

**DOI:** 10.3390/nu18182978

**Published:** 2026-09-11

**Authors:** Alberto Corriero, Rossana Soloperto, Jean-Charles Preiser

**Affiliations:** 1 Department of Interdisciplinary Medicine—ICU Section, University of Bari “Aldo Moro”, Piazza G. Cesare 11, 70124 Bari, Italy; rossana.soloperto@gmail.com; 2 Department of Intensive Care, Université Libre de Bruxelles (ULB), Route de Lennik 808, 1070 Brussels, Belgium; 3 Internal Medicine Department, Institut Jules Bordet—Université Libre de Bruxelles (ULB), 1070 Brussels, Belgium; jean-charles.preiser@hubruxelles.be

## 1. A Collection That Reads as a Timeline

When we launched “Implications of Diet and the Gut Microbiome in Neuroinflammation”, we expected the Special Issue to sort itself by mechanism: a paper on short-chain fatty acids, another on polyphenols, a third on probiotics. We ended up reading it by age instead. Read in sequence, the nine contributions describe one diet–microbiota–neuroinflammation axis observed at successive points along a human life: the microbial ecology of a child’s mouth, the developing brain of a school-age child, the perimenopausal transition, years of established autoimmune or nociplastic disease, the long tail of cancer survivorship, and the hours after acute brain injury.

This was not the organising principle we asked for in the call, and it has proved more useful than the one we had in mind. This axis is not one fixed relationship that holds across a lifetime. The same signal can protect, do nothing, or cause harm, depending on when it meets the host and what state the host is in at that moment.

## 2. A Shared Mechanistic Vocabulary

Despite very different clinical questions, the contributions repeatedly invoke a compact conceptual sequence: dietary and environmental exposures reshape microbial ecology; barrier integrity fails; microbial products (lipopolysaccharide above all) reach the systemic circulation; cytokines, vagal afferents and the hypothalamic–pituitary–adrenal axis carry the signal centrally; and microglia and astrocytes shift towards an activated phenotype [1,2]. The same sequence is traced after traumatic brain injury (TBI) (Contribution 1), and its elements recur across neurological, psychiatric and neurodevelopmental disease (Contribution 2).

Two nodes recur with particular consistency. The first is the short-chain fatty acid (SCFA) pool. Butyrate and propionate are named in several contributions as regulators of epithelial and blood–brain barrier integrity, as histone deacetylase inhibitors, as GPR43/GPR41/GPR109A ligands, and as promoters of regulatory T cell expansion and remyelination in preclinical models (Contributions 1, 2, 3, 5, 6, 7, 9). This relationship is more than correlative: germ-free mice have globally immature microglia, and the same defect appears in animals lacking the SCFA receptor GPR43 [3]. The second node is tryptophan handling. In the menopausal transition, inflammation is proposed to divert tryptophan into the kynurenine pathway and reduce serotonin availability, though the mechanism remains hypothetical (Contribution 3); related tryptophan–serotonin pathways recur in fibromyalgia (Contribution 4) and are supported by disease-specific observations in multiple sclerosis (MS) (Contribution 5). Caries, menopause, MS, cancer and critical illness share no obvious pathology, yet overlapping mediators recur across them.

## 3. The First Window: Ecology Before Language

In children, the axis begins above the stomach. Oral microorganisms may influence gut microbial composition through salivary translocation and immune signalling, but this upstream oral–gut sequence has not been directly shown in children (Contribution 7). The paediatric evidence behind this is thin: trials range from 40 to 262 participants and from one week to ten months, and most measure only one layer of the proposed axis. One result stands out. In 134 extremely preterm infants, *Lactobacillus reuteri* was administered at a dose of 2.5 × 10^8^ colony-forming units (CFU)/day from within 72 h of birth to postmenstrual week 35–36. This treatment left growth and global neurodevelopment unchanged, yet language scores were significantly higher at two years of corrected age. The effect was confined to one domain.

The blind spot reappears at the other end of childhood. Attention-deficit/hyperactivity disorder (ADHD) affects 5–7% of children worldwide, and communication difficulties are among the impairments that most shape daily social function, yet nutrition and microbiome research in ADHD measures attention, hyperactivity and executive function almost exclusively; effects on language and pragmatics are inferred rather than observed (Contribution 8). An intervention acting on one narrow domain may be missed by an instrument built to summarise several.

## 4. Midlife: A Loop That Closes on Itself

The perimenopausal transition is a window in which several risk trajectories intersect (Contribution 3). At age 45, estimated lifetime risk of Alzheimer’s disease is roughly 20% in women against 10% in men. Depressive symptoms are reported in 36.6% of women during perimenopause. Longitudinal cohorts show weight gain of roughly 0.4 to 0.7 kg per year across the transition. These converge in a self-reinforcing loop: disturbed sleep and low mood favour palatable, high-glycaemic intake, which promotes visceral adiposity, dysbiosis and low-grade inflammation, which in turn worsen mood and accelerate amyloid and tau pathology.

A negative result sharpens the picture. A sustained energy gap of 100 kcal/day could be enough to drive this trajectory and would be hard to detect with a food-frequency questionnaire, which may be why the Swiss cohort in that review records no significant change in pastry or added-sugar intake across the transition. The discrepancy may be a measurement problem rather than a biological one: in this window our dietary instruments are less sensitive than the biology we are trying to capture.

## 5. Established Disease and Survivorship

In MS, observational studies return effect sizes rather than directions. Fish or seafood consumption at least weekly, or monthly with fish-oil supplementation, is associated with a 44% reduction in the odds of MS or clinically isolated syndrome, and each energy-adjusted portion of ultra-processed food with an 8% increase in the likelihood of a first demyelinating event (Contribution 5). In experimental autoimmune encephalomyelitis, a 75% carbohydrate diet for six to seven weeks produced earlier onset, higher incidence and pronounced demyelination, while a high-fat canola-oil diet, rich in monounsaturated fatty acids (MUFAs) and about 30% polyunsaturated fatty acids (PUFAs), produced complete protection.

In fibromyalgia, the two nodes reappear: butyrate-producing taxa are depleted, and tryptophan metabolism shifts towards the kynurenine pathway [4]. The link is no longer only correlational: transplanting the microbiota of women with fibromyalgia into germ-free mice induces persistent pain, which resolves once a healthy microbiota is transplanted instead, and an open-label human transplantation trial produced sustained pain relief alongside a measurable shift in the recipients’ own microbiota [4]. Other interventions tried under the same heading—including probiotics, a synbiotic, a Khorasan wheat trial, a low-FODMAP (fermentable oligosaccharide, disaccharide, monosaccharide and polyol) diet, a ketogenic protocol, and a gut-directed enema—remain too heterogeneous to pool (Contribution 4). However, a placebo-controlled probiotic trial did outperform placebo on pain, and larger trials are underway [4]. Whether pain moves with mood and cognition, or separately, is an open question that deserves its own hypothesis.

One candidate lies in the structure of pain itself. Affective and sensory pain thresholds are separable: in major depression, temperatures that are not yet painful are already rated as unpleasant, a bias independent of the sensory pain threshold, a dissociation named emotional allodynia [5,6,7]. If microbiota-directed interventions act on the affective limb of pain rather than on sensory transduction, an unchanged pain score is not a failure but the expected result. Trials should therefore report mood, cognition and pain intensity separately, rather than a composite score that may hide precisely that effect.

Mood and cognition return as endpoints in a very different setting. Cancer engages the axis at every stage of its own trajectory (Contribution 6). Between one-third and one-half of cancers are attributed to modifiable dietary and lifestyle factors, so the exposure long precedes the diagnosis. During treatment, fibre-derived SCFAs are linked to barrier integrity and butyrate to CD8+ T cell cytotoxicity, while diet and microbiome are discussed more broadly as influences on treatment tolerance and on mood. In survivorship, the cognitive and affective sequelae of therapy pose the questions this collection asks of critical illness and of menopause.

## 6. The Acute End: When the Axis Breaks in Hours

Acute brain injury compresses the whole sequence into days. Genus-level microbiota shifts appear within 24 h of experimental TBI, and human intracerebral haemorrhage cohorts show depletion of *Lachnospiraceae*, *Ruminococcaceae* and *Bacteroidaceae* alongside enrichment of *Enterococcaceae* over the first week after admission (Contribution 1). Here the most defensible nutritional recommendations remain restrictive rather than additive: avoid carbohydrate delivery beyond oxidative capacity, about 5 mg/kg/min, in a population where roughly 87% of ICU patients with TBI become hyperglycaemic, and respect the European Society for Clinical Nutrition and Metabolism (ESPEN) range of 0.2–0.3 g/kg/day for supplemental enteral glutamine in critically ill trauma patients.

Timing of nutrient delivery may itself be an intervention (Contribution 9). The gut microbiota oscillates over the day; that oscillation is driven by feeding rhythm rather than by the host clock alone, and disrupting it is sufficient to transfer metabolic disease to germ-free recipients [8]. Restricting intake to part of the day extends the daily fast beyond twelve hours, and longer fasts lower insulin and insulin-like growth factor 1 (IGF-1) signalling, shift metabolism towards ketone bodies and activate autophagy [9]. Continuous 24 h enteral feeding, the ICU default, removes the fasting interval and attenuates that cycle. So far, the mechanism is better supported than the clinical results. Pooled analyses have not settled the question: an early mortality signal did not survive as trials accumulated, while gastrointestinal intolerance persisted in the largest and most recent synthesis. Most of these trials were conducted in general ICU populations rather than dedicated brain-injury cohorts, and none measured neuroinflammation.

## 7. Three Cautions the Collection Earns

Context can invert the sign of a favourable molecule. SCFAs are protective almost everywhere in these pages, yet acetate correlates with worse disability scores in some MS cohorts (Contribution 5). *Akkermansia* is enriched in MS and declines slightly but consistently under a ketogenic diet in the same review, while three months of pasteurised *A. muciniphila* in overweight, insulin-resistant adults improved insulin sensitivity by 29% and lowered markers of liver dysfunction and inflammation [10]. The direction of change in a single taxon is not a therapeutic rule: the same organism can be a marker of disease in one population and a candidate treatment in another.

Metabolic and intermediate clinical outcomes moved together in this trial, but survival did not. In sepsis, a ketogenic protocol produced stable ketosis and, after day 4, no treated patient still required insulin, while 35–60% of controls did; ventilation-free, vasopressor-free and ICU-free days all increased, and 30-day survival was unchanged (Contribution 9). The gap worth watching sits between those intermediate outcomes and survival, not between mechanism and patient benefit.

A named exposure is not a single intervention. “Probiotic”, “fibre” and “SCFA” enter trials as if each were one thing, while the strains and substrates behind those labels are not comparable, and the doses, durations and outcome measures differ from trial to trial, which is why those eleven studies could not be pooled (Contribution 4). The microbiota receiving them differs from one patient to the next. In healthy adults this has been observed prospectively: with 18 adults per arm, a single high-fibre diet split participants into three distinct immunological trajectories, with baseline microbiota diversity differing between the highest- and lowest-inflammation groups, although the primary cytokine outcome was unchanged, while a high-fermented-food diet raised diversity and lowered inflammatory markers across the group [11]. If a single fibre arm splits into responders and non-responders in healthy volunteers, a uniform effect in a ventilated patient or a perimenopausal woman is an optimistic assumption.

## 8. What the Next Generation of Studies Should Look Like

The collection converges on a design brief. Trials should be built around one life stage rather than recruit across all ages and adjust for age afterwards: recruiting preterm infants, women crossing menopause, or ventilated patients after brain injury, and powering the analysis within that group, because effects may vary by life stage and clinical context, a hypothesis this collection raises but does not test directly. They should measure across layers at once, covering diet, oral and gut microbiota, metabolites, barrier function, systemic cytokines, brain biomarkers and cognition, instead of sampling one and inferring the rest. They should treat timing as an experimental variable alongside composition. They should report strain, dose and duration with the precision expected of a drug. And they should pair mechanistic endpoints with patient-centred outcomes, because a metabolome that shifts without a patient who improves is a hypothesis rather than a therapy.

The useful question was never whether a probiotic works, but in whom, on which symptom, and through which microbial change.

## References

[1] Cryan J.F., O’Riordan K.J., Cowan C.S.M., Sandhu K.V., Bastiaanssen T.F.S., Boehme M., Codagnone M.G., Cussotto S., Fulling C., Golubeva A.V. (2019). The microbiota-gut-brain axis. Physiol. Rev..

[2] Sampson T.R., Mazmanian S.K. (2015). Control of brain development, function, and behavior by the microbiome. Cell Host Microbe.

[3] Erny D., Hrabě de Angelis A.L., Jaitin D., Wieghofer P., Staszewski O., David E., Keren-Shaul H., Mahlakoiv T., Jakobshagen K., Buch T. (2015). Host microbiota constantly control maturation and function of microglia in the CNS. Nat. Neurosci..

[4] Varrassi G., Chelidze K., Tran Y.V., Van Pham P.V., Al Alwany A.A., Narváez Encinas M., Sarzi-Puttini P., Myrcik D., Corriero A., Farì G. (2026). The microbiota-gut-brain axis in fibromyalgia: A scoping review. Clin. Exp. Rheumatol..

[5] Strigo I.A., Simmons A.N., Matthews S.C., Craig A.D., Paulus M.P. (2008). Increased affective bias revealed using experimental graded heat stimuli in young depressed adults: Evidence of “emotional allodynia”. Psychosom. Med..

[6] Ushinsky A., Reinhardt L.E., Simmons A.N., Strigo I.A. (2013). Further evidence of emotional allodynia in unmedicated young adults with major depressive disorder. PLoS ONE.

[7] Corriero A., Giglio M., Pilolla A., Galdini F., Mucci O., Vurro M., Fornarelli F., Di Venosa C., Trerotoli P., Puntillo F. (2026). The Emotional Allodynia Questionnaire: Preliminary validation and clinical phenotyping in fibromyalgia. J. Pain Res..

[8] Thaiss C.A., Zeevi D., Levy M., Zilberman-Schapira G., Suez J., Tengeler A.C., Abramson L., Katz M.N., Korem T., Zmora N. (2014). Transkingdom control of microbiota diurnal oscillations promotes metabolic homeostasis. Cell.

[9] Longo V.D., Panda S. (2016). Fasting, circadian rhythms, and time-restricted feeding in healthy lifespan. Cell Metab..

[10] Depommier C., Everard A., Druart C., Plovier H., Van Hul M., Vieira-Silva S., Falony G., Raes J., Maiter D., Delzenne N.M. (2019). Supplementation with *Akkermansia muciniphila* in overweight and obese human volunteers: A proof-of-concept exploratory study. Nat. Med..

[11] Wastyk H.C., Fragiadakis G.K., Perelman D., Dahan D., Merrill B.D., Yu F.B., Topf M., Gonzalez C.G., Van Treuren W., Han S. (2021). Gut-microbiota-targeted diets modulate human immune status. Cell.

